# Pyrrolo[3,2-*d*]pyrimidine Derivatives as Type II Kinase Insert Domain Receptor (KDR) Inhibitors: CoMFA and CoMSIA Studies

**DOI:** 10.3390/ijms13022387

**Published:** 2012-02-22

**Authors:** Xiao-Yun Wu, Wen-Hua Chen, Shu-Guang Wu, Yuan-Xin Tian, Jia-Jie Zhang

**Affiliations:** School of Pharmaceutical Sciences, Southern Medical University, Guangzhou 510515, China; E-Mails: shuguang@fimmu.com (S.-G.W.); tyx523@163.com (Y.-X.T.)

**Keywords:** CoMFA, CoMSIA, KDR inhibitor, pyrrolo[3,2-*d*]pyrimidine derivatives, Surflex-Dock

## Abstract

Kinase insert domain receptor (KDR) inhibitors have been proved to be very effective anticancer agents. Molecular docking, 3D-QSAR methods, CoMFA and CoMSIA were performed on pyrrolo[3,2-*d*]pyrimidine derivatives as non-ATP competitive KDR inhibitors (type II). The bioactive conformation was explored by docking one potent compound **20** into the active site of KDR in its DFG-out inactive conformation. The constructed CoMFA and CoMSIA models produced statistically significant results with the cross-validated correlation coefficients *q*^2^ of 0.542 and 0.552, non-cross-validated correlation coefficients *r*^2^ of 0.912 and 0.955, and predicted correction coefficients *r*^2^_pred_ of 0.913 and 0.897, respectively. These results ensure the CoMFA and CoMSIA models as a tool to guide the design of a series of new potent KDR inhibitors.

## 1. Introduction

Angiogenesis, the form of new blood vessels by capillary sprouting from pre-existing vasculatures, is a normal process for organ development during embryogenesis, wound healing and female reproductive cycling [1,2]. Abnormal regulation of angiogenesis has been shown to get involved in many diseases, such as diabetic retinopathy, psoriasis, rheumatoid arthritis, and cancer. In particular, it is widely recognized that the growth and metastasis of solid tumors is dependent on angiogenesis [3,4]. Out of the many factors that are involved in angiogenesis, vascular endothelial growth factors (VEGFs) are of particular interests [5,6]. The VEGFs are required for vasculogenesis and angiogenic sprouting, and act through receptor tyrosine kinase (VEGFR-1, -2, and -3). Among them, VEGF receptor-2 (VEGFR-2) or kinase insert domain receptor (KDR) play crucial roles in vessel sprouting and new vessel initiation in early stage of angiogenesis.

It is well known that inhibiting of KDR leads to suppression of angiogenesis and tumor growth. A number of preclinical and clinical studies have shown that many small-molecule KDR inhibitors are capable of inhibiting angiogenesis, tumor progression, and dissemination [7–11]. The vast majority of the KDR inhibitors known to date, such as Gefitinib, are ATP-competitive and classified as type I inhibitors. Such inhibitors target the ATP binding pocket in its active conformation of the activation loop. This conformation is normally referred to as DFG “in” based on the position of the conserved triad aspartate-phenylalanine-glycine (DFG) at the entrance of the activation loop. Type I inhibitors typically function in the DFG “in” conformation of KDR through hydrogen bonding with the backbone residues of the hinge region as well as hydrophobic interactions in and around the adenine region. These features, however, make it quite difficult to design highly selective type I inhibitors [12,13]. In addition, ATP-competitive inhibitors have to compete with high levels of intracellular ATP, leading to a significant difference between the *in vitro* and *in vivo* activities. In response to these issues, non-ATP competitive kinase inhibitors, such as Imatinib have been identified. These inhibitors, *i.e.*, type II inhibitors bind to and stabilize an inactive kinase form that features the DFG motif in an “out” conformation. The different position of the DFG residues in the “out” form creates a new hydrophobic binding pocket that is adjacent to the ATP-binding site. This pocket, also known as the allosteric site, is characteristic of kinase in an inactive conformation. Type II inhibitors predominantly occupy the ATP binding site, but they also exploit unique hydrogen bonding and hydrophobic interactions with the allosteric site. Compared with type I kinase inhibitors, type II inhibitors have several advantages, including great cellular potency and improved kinase selectivity. In addition, type II inhibitors, because of their interactions with both the ATP pocket and the allosteric site, may provide an avenue to overcome the mutations that induce resistance to the other types of inhibitors [14–18].

Recently, Oguro *et al.* reported a set of pyrrolo[3,2-*d*]pyrimidine derivatives **1**–**52** (Table 1) as potent and selective KDR inhibitors in biochemical and cellular assays [19,20]. These compounds are structurally distinct from the ATP-competitive inhibitors, and the X-ray crystallographic analysis has shown that compound **27** induces an inactive conformation, in which the diphenylurea moiety occupies the hydrophobic pocket created by the conformation change of DFG motif (DFG-out) [19,20]. In the present study, to gain further insight into the interactions of these compounds with the KDR, we employed the molecular docking-guided three dimensional quantitative structure-activity relationship (3D-QSAR) study, comparative molecular field analysis (CoMFA) [21] and comparative molecular similarity indices analysis (CoMSIA) [22] to address how steric, electrostatic, hydrophobic and hydrogen-bonding interactions modulate the inhibitory activities.

## 2. Materials and Methods

### 2.1. General

The crystallographic coordinates of KDR in complex with small-molecule inhibitors were obtained from the Brookheaven Protein Databank as entries 2OH4 [23]. All the molecular modeling and calculations were performed using Sybyl 7.3 molecular modeling package [24].

### 2.2. Data Sets

In this study, 52 pyrrolo[3,2-*d*]pyrimidine derivatives were taken from the work of Oguro *et al.* [19,20]. Their structures and inhibitory activities are listed in Tables 1 and 2. The IC_50_ values (M) were converted to the corresponding pIC_50_ (= −logIC_50_). On the basis of the diversity in the structures and activities, these 52 compounds were divided into two groups: 44 compounds were used as the training set to build the 3D-QSAR models, and 8 compounds that are marked with an asterisk in Table 1 were used as the test set to evaluate the predictive power of the developed CoMFA and CoMSIA models.

### 2.3. Molecular Docking

Because the crystal structure of KDR in complex with pyrrolo[3,2-*d*]pyrimidine was not available in the Brookheaven Protein Databank (PDB), the bioactive conformation was simulated by docking using Surflex-dock program. The crystallographic coordinates of KDR in complex with its inhibitor, which was reported to be in the inactive DFG-out conformation of KDR, were obtained from the PDB as entries 2OH4 [23]. Surflex-Dock program [25,26] has been widely used to calculate the protein–ligand interactions, and to efficiently predict the active conformations [27–35]. Surflex-Dock uses a Protomol-based method and an empirical scoring function to dock a ligand into the binding site of a receptor. The Protomol is an idealized representation of a ligand that forms every potential interaction with the binding site. Surflex-Dock’s scoring function contains the factors that play crucial roles in the ligand-receptor interaction, including hydrophobic, polar, repulsive, entropic and solvation terms. In this study, the Protomol was generated using a ligand-based approach. During the Protomol generation process, two particular parameters, *Protomol_bloat* and *Protomol_threshold*, were specified to form the appropriate binding pocket. The former determines how far the site should extend from a potential ligand, whereas the latter determines how deep the atomic probes that are used to define the Protomol can penetrate into the protein. In the present work, *Protomol_bloat* and *Protomol_threshold* default values (0 and 0.5, respectively) were used when a reasonable binding pocket was obtained. During the docking process, the default values of all the other parameters were assigned. The highest-scored conformation of a potent compound **20** based on the Surflex-Dock scoring functions, was selected as the final bioactive conformation.

### 2.4. Molecular Modeling

In the 3D-QSAR study, the selection of active conformations is a key step for CoMFA and CoMSIA studies. The bioactive conformation of compound **20** was simulated using Surflex-Dock. The docked conformation with the highest total score was used as the template to construct the 3D structures of the rest compounds in the data set. Structural energy minimization process was performed using the Tripos force field with a distance-dependent dielectric and Powell gradient algorithm with a convergence criterion of 0.001 kcal/mol. Partial atomic charges were calculated using Gasteiger-Hückel method.

### 2.5. Molecular Alignment

**Figure 1:**
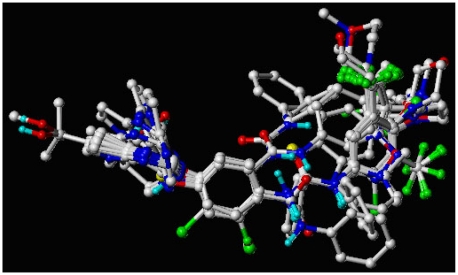
Superimposition of compounds in the training and test set.

In the 3D-QSAR study, the alignment rule is also a key step. The predictive accuracy of the CoMFA and CoMSIA models and the reliability of the contour maps are directly dependent on the structural alignment rule. The compounds were aligned by the atomfit to the template **20**. The aligned compounds are shown in Figure 1.

### 2.6. CoMFA and CoMSIA Studies

Standard CoMFA and CoMSIA procedures were performed. A 3D cubic lattice was created automatically by extending at least 4 Å beyond all the aligned molecules in *X*, *Y* and *Z* directions with 2.0 Å grid spacing. The CoMFA steric (Lennard-Jones potential) and electrostatic (Coulomb potential) fields at each lattice were calculated using the standard Tripos force field method. A distance dependent dielectric constant of 1.0 was used, and an sp^3^ hybridized carbon atom with one positive charge and a radius of 1.52 Å served as a probe atom to calculate the steric and electrostatic fields. The default cutoff value of 30.0 kcal/mol was adopted.

Compared with CoMFA, CoMSIA methodology has the advantage of exploring the impacts of more fields. In addition to the steric (S) and electrostatic (E) fields used in CoMFA, the CoMSIA method defines hydrophobic (H), hydrogen bond donor (D), and hydrogen bond acceptor (A) descriptors. The CoMSIA fields were derived, according to Klebe *et al.* [22], from the same lattice box that was used in the CoMFA calculations, with a grid spacing of 2 Å and a probe carbon atom with one positive charge and a radius of 1.0 Å as implemented in Sybyl. Arbitrary definition of cutoff limits was not required in CoMSIA method, wherein the abrupt changes of potential energy near the molecular surface were taken into account in the distance dependent Gaussian type functional form. The default value of 0.3 was used as the attenuation factor.

### 2.7. PLS Regression Analysis and Validation of QSAR Models

Partial least squares (PLS) approach was used to derive the 3D QSAR models. The CoMFA and CoMSIA descriptors were used as independent variables and the pIC_50_ values were used as dependent variables. CoMFA and CoMSIA column filtering was set to 2.0 kcal/mol to improve the signal-to-noise ratio. The leave-one-out (LOO) cross-validation was carried out to obtain the optimal number of components (N) and the correlation coefficient *q*^2^. The obtained N was then used to derive the final QSAR model and to obtain the non-cross-validation correlation coefficient *r*^2^, standard error of estimate (SEE), and Fischer (F) ratio value.

To assess the predictive power of the derived 3D-models, a set of test compounds that had known biological activities, was used to validate the obtained models. The predictive correlation *r*^2^_pred_ value was calculated using

$${r^{2}}_{\text{pred}}=(\text{SD}-\text{PRESS})/\text{SD}$$

Wherein SD is the sum of the squared deviations between the biological activities of the test compounds and the mean activities of the training compounds, and PRESS is the sum of the squared deviations between the experimental and the predicted activities of the test compounds.

## 3. Results and Discussion

### 3.1. Binding Modes of Pyrrolo[3,2-d]pyrimidine Derivatives

**Figure 2:**
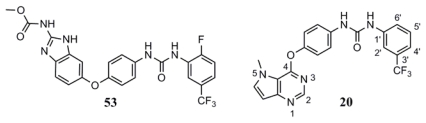
Compound **53** from 2OH4 and atom-numbered compound **20**.

**Figure 3:**
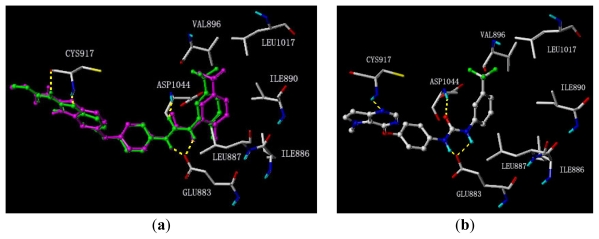
Binding conformations of (**a**) co-crystallized (magenta) and re-docked (green) **53** and (**b**) docked compound **20** at the active site of kinase insert domain receptor (KDR) in the inactive conserved triad aspartate-phenylalanine-glycine (DFG)-out conformation. Key residues are displayed and hydrogen bonds are displayed in dotted lines.

To determine the probable binding conformations of these compounds, Surflex-Dock was used to dock one potent compound **20** into the active site of KDR (PDB code: 2OH4). First, the docking reliability was validated by a known inhibitor **53** (Figure 2) that was reported to bind in the DFG-out inactive conformation of KDR [23]. The co-crystallized **53** was re-docked into the binding site, and the docked conformation having the highest total score was selected as the most probable binding conformation (Figure 3). The low root mean-square deviation (RMSD) of 0.58 Å between the docked and the crystal conformations demonstrated the high reliability of Surflex-dock in reproducing the experimentally observed binding mode for these KDR inhibitors. As shown in Figure 3a, redocked **53** was almost in the same orientation with co-crystallized **53** at the active site of KDR. Therefore, Surflex-Dock docking protocol and the used parameters were extended in search for the binding conformations of KDR inhibitors.

The binding mode of compound **20** at the active site of KDR in its inactive conformation is shown in Figure 3b. It is clear that compound **20** adopts an overall conformation that is very similar to that of compound **53**, and forms multiple hydrogen bonds with KDR. Specifically, the 1-nitrogen of the pyrrolo[3,2-*d*]pyrimidine subunit forms one hydrogen bond with the backbone-NH of Cys917 in the hinge region of KDR at the angle and distance of 130.76° and 3.253 Å, respectively. In the allosteric site, the two NH groups of the urea moiety form two hydrogen bonds with the side chain carboxylate of Glu883 at the angles of 99.12° and 97.85°, and the distances of 2.566 Å and 2.873 Å, respectively, whereas the CO moiety interacts with the backbone-NH of Asp1044 in the DFG motif through hydrogen bonding at the angle and distance of 151.02° and of 2.666 Å, respectively. These interactions are typical characteristics of the interactions between the inhibitors and the inactive conformation of the kinase. In addition, the terminal phenyl moiety occupies the hydrophobic pocket that is formed from residues Ile886, Leu887, Ile890, Val896 and Leu1017.

### 3.2. CoMFA and CoMSIA Results

**Figure 4:**
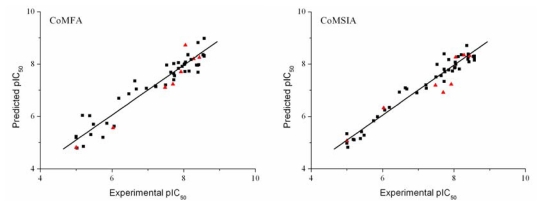
Graphs of the experimental *versus* predicted pIC_50_ values of the training (■) and test (▴) compounds from the CoMFA and CoMSIA models.

The CoMFA and CoMSIA 3D-QSAR models were derived from a training set of 44 compounds. The statistical results of the CoMFA and CoMSIA 3D-QSAR models are presented in Table 3. The CoMFA model gave a cross-validated correlation coefficient *q*^2^ of 0.542, an optimal number of principal components (N) of 4 and a non-cross-validated correlation coefficient *r*^2^ of 0.912. The corresponding contributions of steric and electrostatic fields were 52.5% and 47.5%, respectively. The CoMSIA model gave a cross-validated correlation coefficient *q*^2^ of 0.552, an optimal number of principal components of 5 and a non-cross-validated correlation coefficient *r*^2^ of 0.955. The corresponding contributions of steric, electrostatic, hydrophobic, hydrogen bond donor and acceptor fields were 18.4%, 22.8%, 34.3%, 6.3% and 18.2%, respectively. Both the CoMFA and CoMSIA models were satisfactory from the viewpoint of statistical significance. The activities of the 44 training compounds were predicted with the constructed CoMFA and CoMSIA models. The predicted pIC_50_ values are shown in Table 2 and Figure 4. It can be seen that the predicted pIC_50_ values were in good agreement with the experimental values, indicating that the obtained CoMFA and CoMSIA models had strong predictive ability.

### 3.3. Validation of the 3D-QSAR Models

The predictive powers of the CoMFA and CoMSIA models were validated by the eight test compounds. The predicted pIC_50_ values were found to be in good agreement with the experimental data within an acceptable error range (Table 2 and Figure 4). The predictive correction coefficients of the CoMFA and CoMSIA models were 0.913 and 0.897, respectively. This result indicates that the CoMFA and CoMSIA models may be used to predict the inhibitory activities of novel pyrrolo[3,2-*d*]pyrimidine derivatives as type-II KDR inhibitors.

### 3.4. Contour Analysis

**Figure 5:**
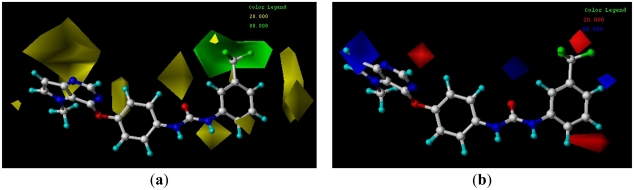
CoMFA STDEV*COEFF contour maps. (**a**) Favorable (green) and unfavorable (yellow) steric fields. (**b**) Electropositive (blue) and electronegative (red) fields. Compound **20** was overlaid in each map.

**Figure 6:**
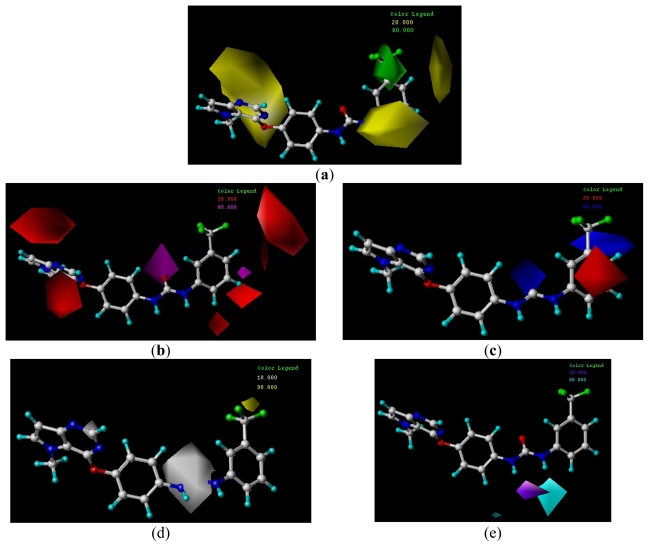
STDEV*COEFF contour maps. (**a**) Favorable (green) and unfavorable (yellow) steric fields. (**b**) Electropositive (blue) and electronegative (red) fields. (**c**) Favorable (yellow) and unfavorable (gray) hydrophobic fields. (**d**) Favorable (cyan) and unfavorable (purple) hydrogen bond donor fields. (**e**) Favorable (magenta) and unfavorable (red) hydrogen bond acceptor fields. Compound **20** was overlaid in each plot.

To visualize the results of the CoMFA and CoMSIA models, the 3D coefficient contour maps were generated. The CoMFA and CoMSIA results were graphically interpreted by the field contribution maps using the STDEV*COEFF field type. The contour maps of CoMFA (steric and electrostatic) and CoMSIA (steric, electrostatic, hydrophobic, hydrogen bond donor and acceptor fields) are shown in Figures 5 and 6, respectively. Compound **20** was displayed in the map in aid of visualization. All the contours represented the default 80% and 20% level contributions for favorable and unfavorable regions, respectively, except 90% and 10% level contributions in Figure 6c, respectively.

#### 3.4.1. CoMFA Contour Maps

The CoMFA contour maps of the steric and electrostatic fields are shown in Figure 5. In the map of steric field, the green contours represent the regions in which bulky groups confer an increase in the activity, whereas the yellow ones represent the regions where bulky groups may lead to a decrease in the activity. Similarly, in the map of electrostatic field, the blue contours indicate the regions where electropositive substitution increases the inhibitory activity, whereas the red contours indicate the regions where electronegative substitution increases the activity.

In the CoMFA steric contour map (Figure 5a), a large green contour near the 3′-position of the terminal 3′-trifluoromethylphenyl group of compound **20** suggests that introducing of bulky groups at this position would increase the activity. In consistent with this, compounds bearing bulky groups at this position, for example, compounds **18**, **20**, **21** and **23** showed high activities, whereas the ones bearing small groups at the same position, for example compounds **16** and **22**, showed low activities. Large yellow contours near the 4′- and 5′-positions of the terminal 3′-trifluoromethylphenyl group suggest that introducing of bulky groups at these positions would decrease the activity. This is in agreement with the fact that compounds **44**–**52** with bulky groups at 4′- or 5′-positions of the terminal 3′-trifluoromethylphenyl showed decreased activities. In addition, big yellow contours near the pyrrolo[3,2-d]pyrimidine subunit suggest that steric bulkiness is unfavorable by the model. When pyrrolo[3,2-d]pyrimidine subunit is conjugated to the central phenyl group via a sulfur or nitrogen atom (X), the subunit falls into the region of the yellow contour, which suggests that a decrease in the inhibitory activity will be observed. For example, sulfur-linked derivative **12** (IC_50_ = 110 nM) and amine-linked derivative **13** (IC_50_ = 1400 nM) were 3- and 40-fold less active than oxygen-linked derivative **16** (IC_50_ = 33 nM), respectively.

In the CoMFA electrostatic contour map, a small red contour near the 3′-position of the 3′-trifluoromethylphenyl group of compound **20** indicates that introduction of electronegative groups around this position would increase the inhibitory activity. This, together with the green contour discussed above, suggests that electronegative and bulky groups near the 3′-position of the terminal phenyl group are favored by the CoMFA model. A blue contour near the 4′-position of the 3′-trifluoromethylphenyl group indicates that introducing of electropositive groups around this position would increase the inhibitory activity. For example, compound **24** with electropositive hydrogen at the 4′-position showed higher activity than the corresponding compounds **45**–**48** with electronegative substituents.

#### 3.4.2. CoMSIA Contour Maps

The CoMSIA steric and electrostatic contour maps of compound **20** are shown in Figure 6a,b, respectively. These contours are quite similar to those of CoMFA. Therefore, our following discussion will focus on the hydrophobic, hydrogen bond donor and acceptor fields.

Figure 6c shows the hydrophobic contour maps in which yellow and gray contours indicate the regions where hydrophobic and hydrophilic groups are favored by the model, respectively. A yellow contour near the 3′-position of the terminal 3′-trifluoromethylphenyl group indicates that hydrophobic substituent at this position would increase the activity. This has been noticed in compounds **18**, **20**, **21** and **23** bearing hydrophobic Cl, CF_3_, Br and CH_3_, respectively. These compounds exhibited increased activities. This hydrophobic interaction may play a crucial role in improving of the binding affinity, since it is also observed in the CoMFA and CoMSIA steric contour maps. The large gray contour near the urea indicates that hydrophilic urea at this position is favorable.

The CoMSIA hydrogen bond donor and acceptor contour plots are shown in Figure 6d,e respectively. The cyan contours represent the regions where hydrogen bond-donating groups increase the activity, whereas the purple contours represent the regions where hydrogen bond-donating groups decrease the activity. Similarly, the magenta contours indicate the regions where hydrogen bond-accepting groups increase the inhibitory activity, whereas the red contours indicate the regions where hydrogen bond-accepting groups decrease the activity.

The cyan contours near the NH of urea indicate that hydrogen bond-donating groups are favored. This is well consistent with the observations that NH group in this region forms stable hydrogen bonds with the residue of Glu883 as hydrogen bond donor, and that the amide derivatives **6** and **7** having a CH_2_ to replace the NH of the urea **16** led to more than 300- and 100-fold decreases in the activities, respectively. A large magenta contour located on the carbonyl oxygen of the urea moiety suggests that hydrogen bond-accepting groups are favored in this region. This is evident from the fact that thiourea derivative **8** was about 100-fold less active than the corresponding urea derivative **16**, and that the carbonyl oxygen of the urea moiety was engaged in hydrogen bonding interaction with the Asp1044 in the DFG motif.

### 3.5. Design of New Inhibitors

As shown above, the CoMFA and CoMSIA have provided detailed insight into the key structural requirements for potent activities of the inhibitors of this class. Specifically, the urea plays a crucial role in the inhibitory activity—its replacement with thiourea or with an acetamide leads to a complete loss of the activity. Substituting of the oxygen linker with sulfur or nitrogen atoms affords less active compounds. Introducing of appropriately bulky and strongly hydrophobic groups at the 3′-position of the terminal phenyl group may significantly increase the activity. To demonstrate the practical values of these structure-activity relationships, a series of new inhibitors were designed and their pIC_50_ values were predicted with the established CoMFA and CoMSIA models (Table 4).

## 4. Concluding Remarks

In this study, 3D-QSAR analyses, CoMFA and CoMSIA, have been applied to a set of recently synthesized pyrrolo[3,2-*d*]pyrimidine derivatives as type II KDR inhibitors. The binding mode of the template molecule **20** was clarified by Surflex-dock. The CoMFA and CoMSIA models showed statistically significant results in terms of cross-validated coefficients and conventional coefficients. Their predictive capabilities were verified by the test compounds. The derived CoMFA and CoMSIA models showed predictive cross-validated coefficients of 0.913 and 0.897, respectively, and the activities of the training and test compounds were predicted with good accuracy. Based on the obtained structure-activity relationships, a series of new inhibitors were designed to have excellent activities that were predicted with the developed CoMFA and CoMSIA models. Thus, these models may be expected to serve as a tool to guide the future rational design of pyrrolo[3,2-*d*]pyrimidine-based novel type II KDR inhibitors with potent activities.

## Figures and Tables

**Table 1:** Structures of compounds **1**–**52**.

Structure	Compound	Substituent	

		R_1_	R_2_
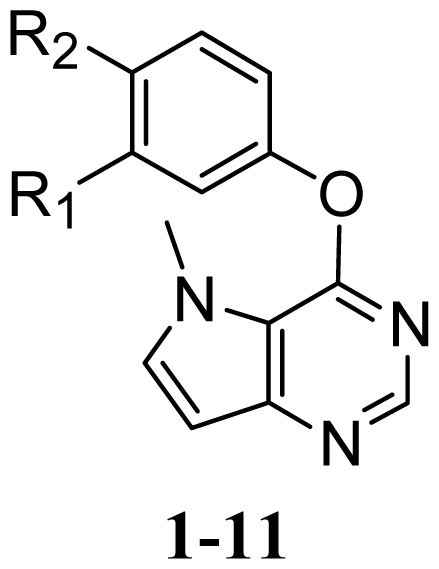	**1**	NHCONHPh	H
	2^*^	NHCOPh	H
	**3**	CONHPh	H
	**4**	H	NHCOPh
	**5**	H	CONHPh
	6^*^	H	NHCOCH_2_Ph
	**7**	H	CH_2_CONHPh
	**8**	H	NHCSNHPh
	**9**	H	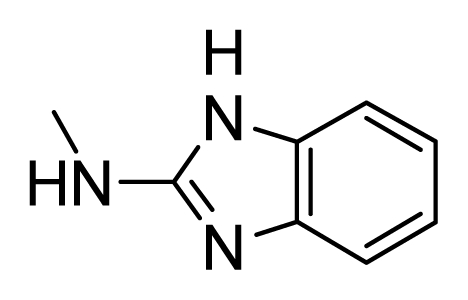
	**10**	H	NHCONHMe
	**11**	H	NHCONHPr
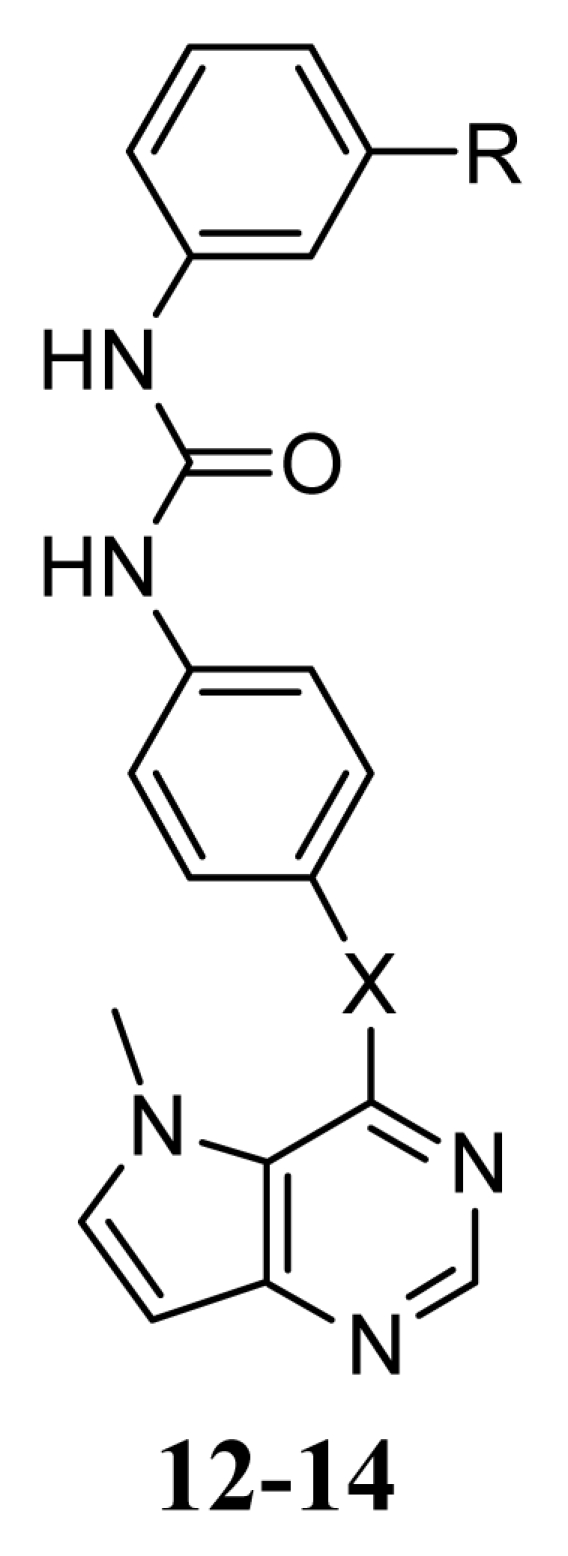		X	R
	**12**	S	H
	**13**	NH	H
	**14**	N(Me)	3-CF_3_
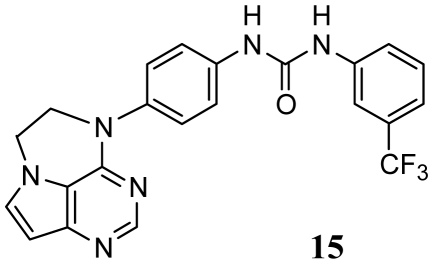			

Structure	Compound	Substituent				

		R	R_1_	R_2_	R_3_	R_4_
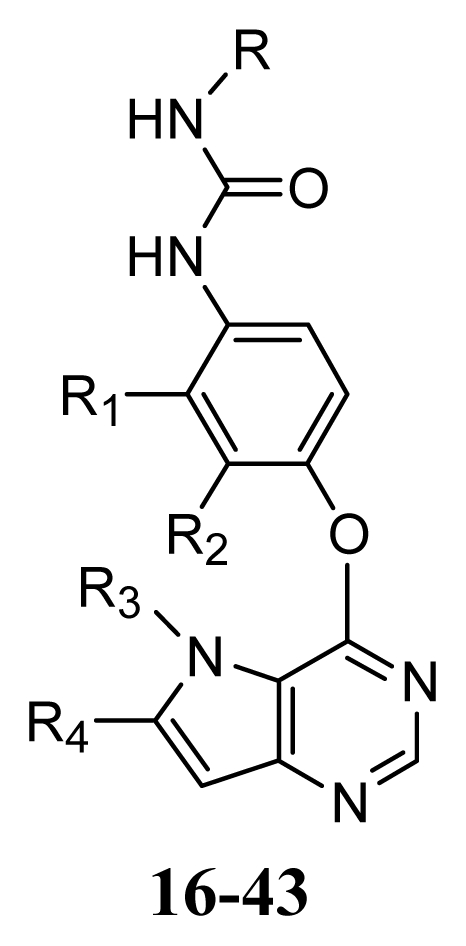	16*	Ph	H	H	Me	H
	**17**	2-ClPh	H	H	Me	H
	**18**	3-ClPh	H	H	Me	H
	**19**	4-ClPh	H	H	Me	H
	20*	3-CF_3_Ph	H	H	Me	H
	**21**	3-BrPh	H	H	Me	H
	**22**	3-FPh	H	H	Me	H
	**23**	3-MePh	H	H	Me	H
	24*	3-CF_3_Ph	Cl	H	Me	H
	**25**	3-CF_3_Ph	H	Cl	Me	H
	**26**	3-CF_3_Ph	OMe	H	Me	H
	**27**	3-CF_3_Ph	F	H	Me	H
	**28**	3-CF_3_Ph	Cl	H	(CH_2_)_2_OH	H
	29*	3-CF_3_Ph	Cl	H	(CH_2_)_2_OMe	H
	**30**	3-CF_3_Ph	Cl	H	(CH_2_)_2_O(CH_2_)_2_OH	H
	**31**	3-CF_3_Ph	Cl	H	Me	CH_2_OH
	**32**	3-CF_3_Ph	Cl	H	Me	CH_2_OMe
	**33**	3-CF_3_Ph	Cl	H	Me	CMe_2_OH
	**34**	2-C_5_H_4_N	Cl	H	Me	H
	**35**	3-C_5_H_4_N	Cl	H	Me	H
	**36**	4-C_5_H_4_N	Cl	H	Me	H
	**37**	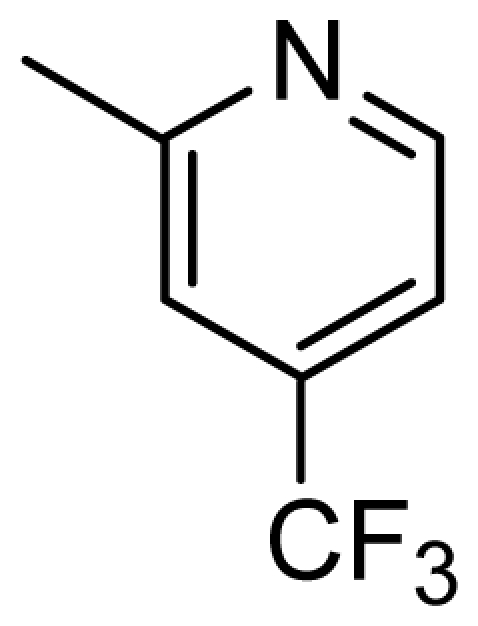	Cl	H	Me	H
	**38**	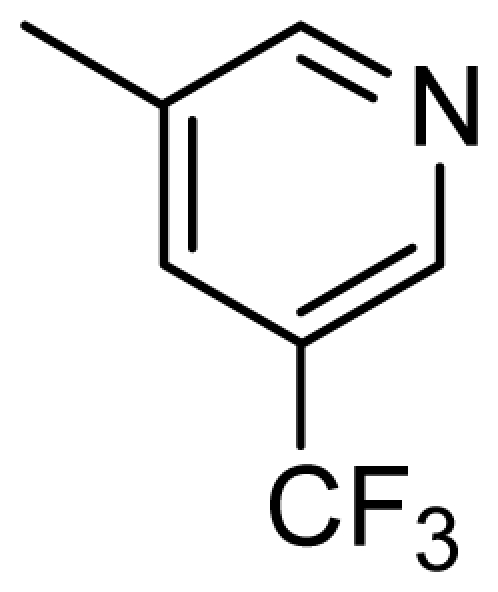	Cl	H	Me	H
	**39**	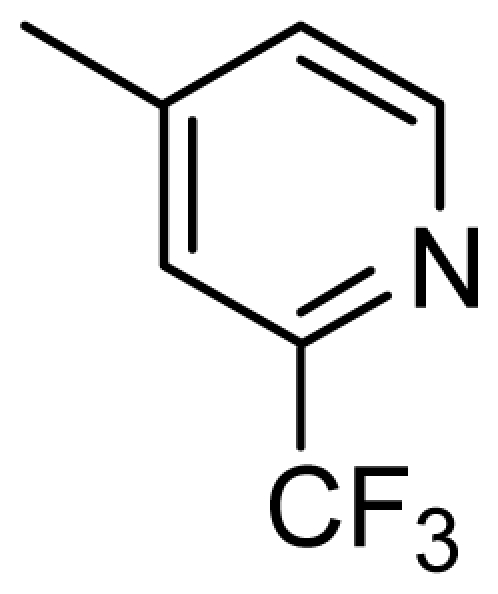	Cl	H	Me	H
	**40**	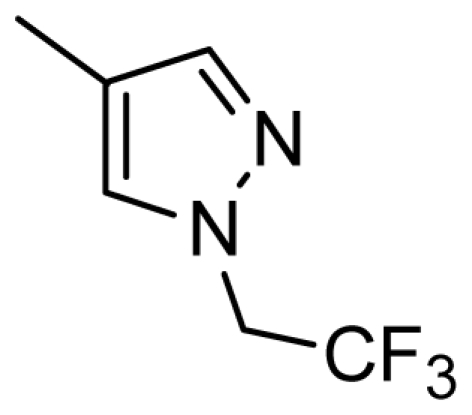	Cl	H	Me	H
	**41**	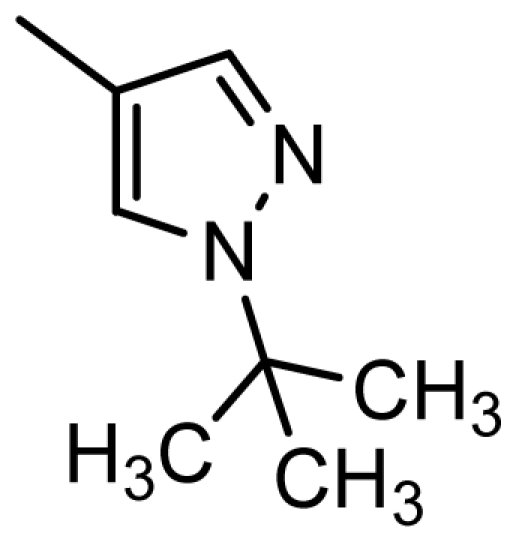	Cl	H	Me	H
	**42**	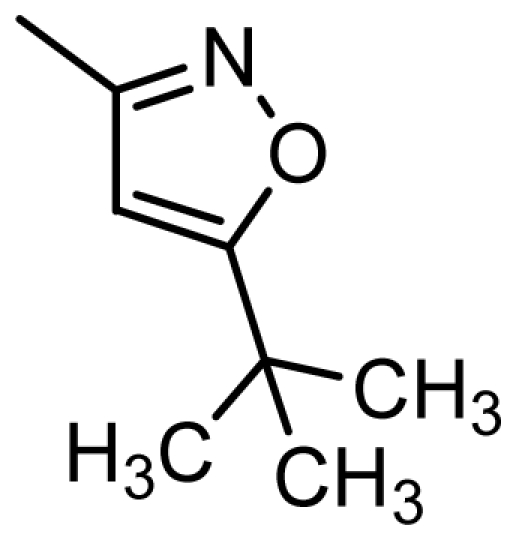	Cl	H	Me	H
	**43**	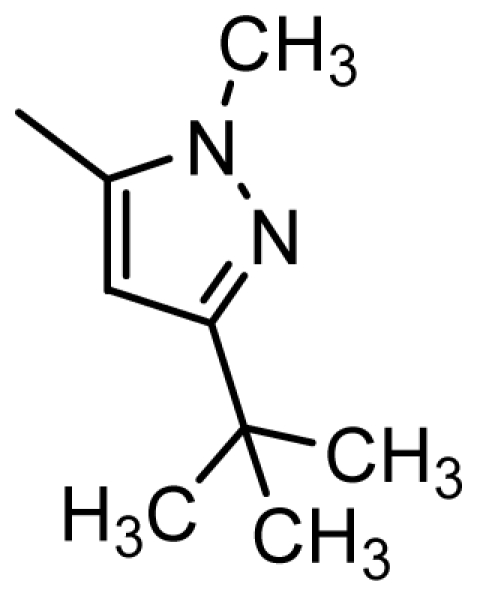	Cl	H	Me	H
		**R_1_**			**R_2_**	
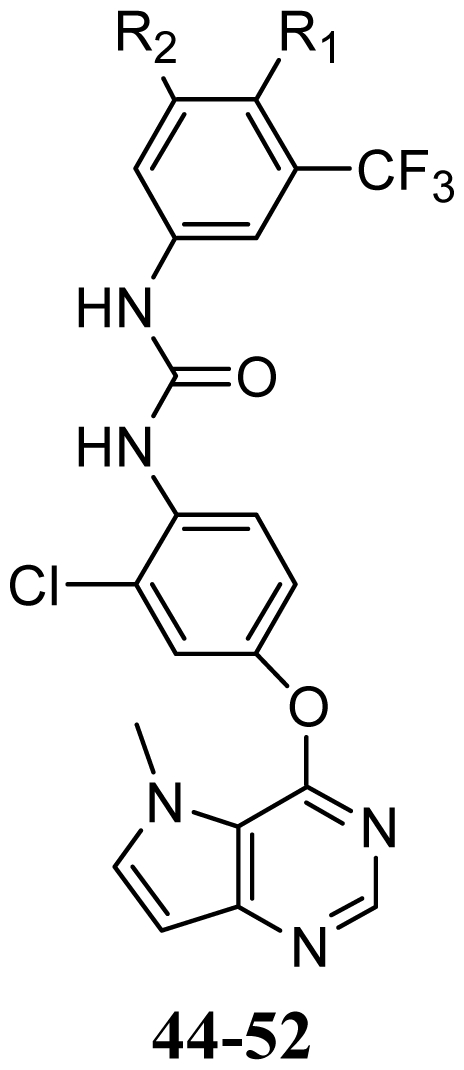	**44**	OMe			H	
	**45**	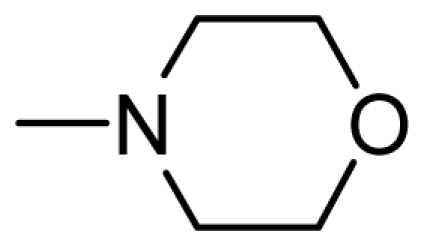			H	
	**46**	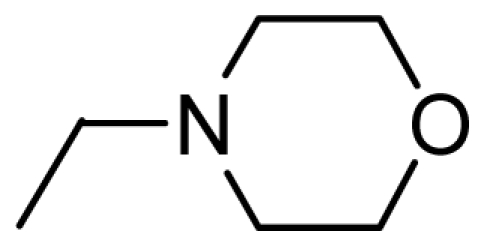			H	
	**47**	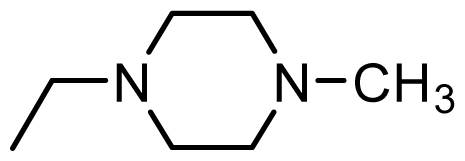			H	
	48*	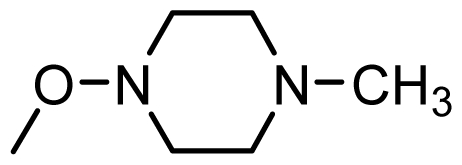			H	
	49*	H			OMe	
	**50**	H			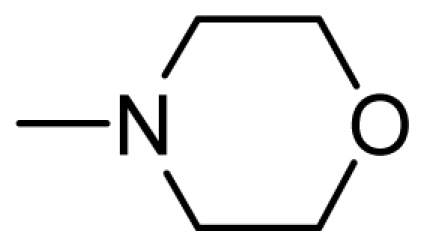	
	**51**	H			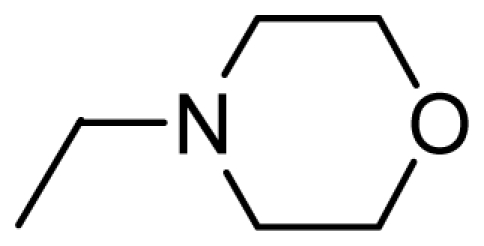	
	**52**	H			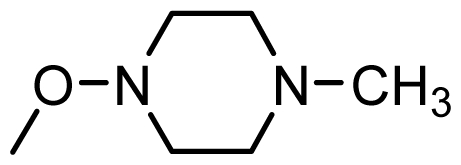	

* Test set.

**Table 2:** The experimental pIC_50_, predicted pIC_50_ and their residuals of compounds **1**–**52**.

Compound	Exp pIC_50_	COMFA		COMSIA	

		Pred	Resid	Pred	Resid
**1**	5.745	5.194	0.551	5.834	−0.089
2*	6.027	5.563	0.464	6.327	−0.300
**3**	6.060	5.621	0.439	6.246	−0.185
**4**	5.027	4.788	0.239	4.821	0.206
**5**	5.000	5.192	−0.192	4.980	0.020
6*	5.000	4.808	0.192	5.074	−0.074
**7**	5.481	5.698	−0.216	5.285	0.197
**8**	5.174	6.042	−0.868	5.129	0.045
**9**	5.377	6.029	−0.652	5.156	0.221
**10**	5.201	4.852	0.349	5.108	0.093
**11**	5.409	5.307	0.102	5.420	−0.011
**12**	6.959	7.074	−0.116	6.895	0.063
**13**	5.854	5.738	0.116	5.987	−0.133
**14**	5.000	5.233	−0.233	5.341	−0.341
**15**	8.149	7.729	0.420	7.810	0.339
16*	7.481	7.103	0.379	7.189	0.293
**17**	6.469	6.863	−0.394	6.927	−0.458
**18**	8.387	7.959	0.428	8.187	0.200
**19**	7.495	7.201	0.293	7.503	−0.008
20*	8.276	8.209	0.066	8.341	−0.066
**21**	8.357	7.969	0.387	8.710	−0.354
**22**	7.721	7.400	0.321	7.335	0.387
**23**	8.569	8.323	0.246	8.153	0.415
24*	8.432	8.255	0.177	8.324	0.108
**25**	7.523	7.966	−0.443	7.987	−0.464
**26**	7.854	8.056	−0.202	8.171	−0.317
**27**	8.208	8.176	0.032	8.357	−0.149
**28**	8.398	8.812	−0.414	8.386	0.012
29*	8.046	8.717	−0.671	8.262	−0.216
**30**	8.569	8.977	−0.408	8.308	0.261
**31**	8.013	7.987	0.027	7.851	0.162
**32**	7.854	7.835	0.019	7.774	0.080
**33**	7.745	7.554	0.191	7.701	0.044
**34**	6.678	7.047	−0.369	7.042	−0.364
**35**	6.638	7.354	−0.716	7.090	−0.451
**36**	6.187	6.691	−0.504	6.342	−0.155
**37**	8.569	8.307	0.262	8.265	0.303
**38**	8.538	8.357	0.181	8.288	0.249
**39**	8.420	7.983	0.438	8.084	0.336
**40**	8.398	7.689	0.709	8.273	0.125
**41**	8.119	8.357	−0.238	8.282	−0.163
**42**	8.051	8.079	−0.028	7.993	0.058
**43**	7.721	8.019	−0.297	8.392	−0.670
**44**	8.027	8.013	0.013	7.875	0.152
**45**	7.215	7.165	0.050	7.203	0.011
**46**	7.721	7.634	0.088	7.805	−0.084
**47**	8.032	7.721	0.310	8.072	−0.041
48*	7.921	7.706	0.215	7.226	0.695
49*	7.699	7.234	0.465	6.911	0.788
**50**	7.222	7.135	0.087	7.073	0.149
**51**	7.638	7.689	−0.051	7.553	0.086
**52**	7.959	7.914	0.044	7.735	0.223

* Test set.

**Table 3:** Statistical parameters for the CoMFA and CoMSIA models.

	*N*	*q*^2^	*r*^2^	*SEE*	*F*	*r*^2^_pred_	Field contribution				

							S	E	H	D	A
CoMFA	4	0.542	0.912	0.376	100.462	0.913	0.525	0.475	-	-	-
CoMSIA	5	0.552	0.955	0.272	161.245	0.897	0.184	0.228	0.343	0.063	0.182

*q*^2^: Cross-validated correlation coefficient; *r*^2^: non-cross-validated correlation coefficient; *r*^2^_pred_: predictive correlation coefficient; *SEE*: standard error of estimate; *F*: Fischer ratio; *N*: optimal number of principal components; S: steric field; E: electrostatic field; H: hydrophobic field; D: hydrogen bond donor field; A: hydrogen bond acceptor field.

**Table 4:** Structures and predicted pIC_50_ values of newly designed derivatives.

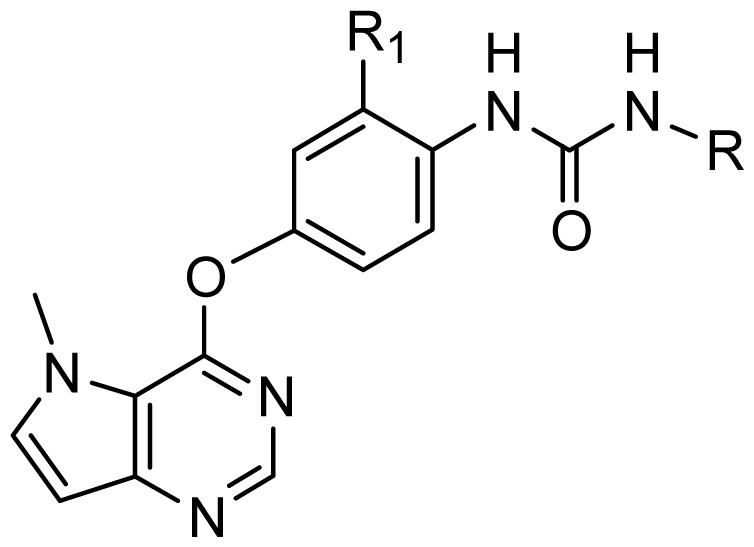				

**Compound**	**R**	**R_1_**	**Predicted pIC_50_**	

			CoMFA	CoMSIA
D1	Cl	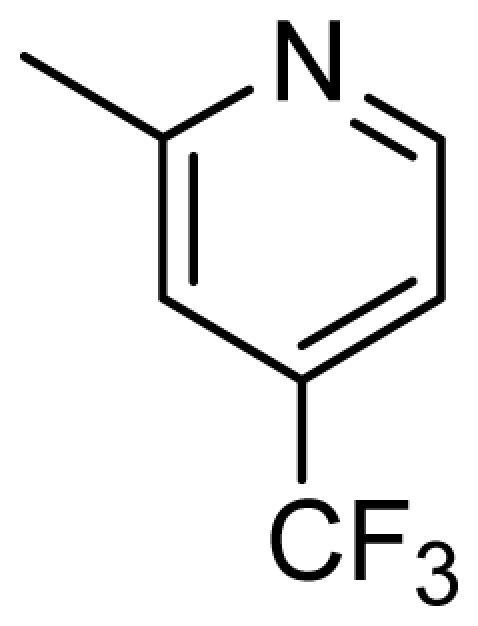	8.162	8.052
D2	Cl	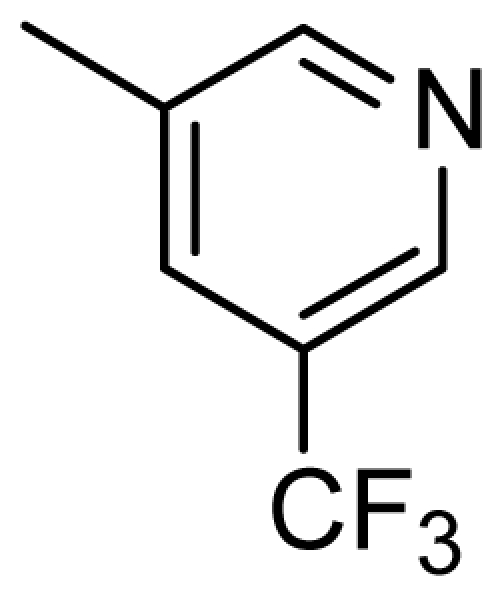	8.463	8.049
D3	Cl	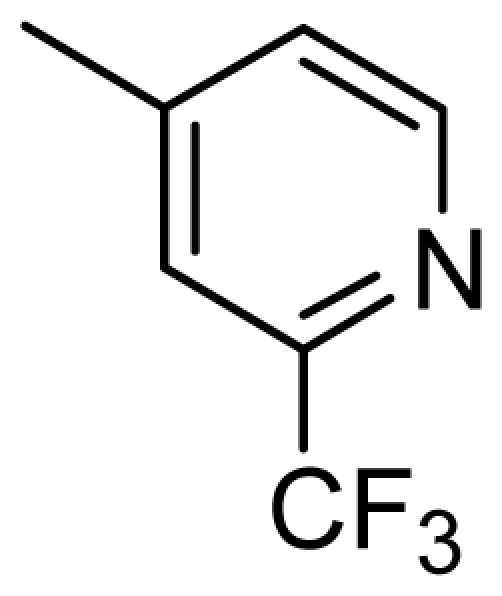	7.972	7.876
D4	Cl	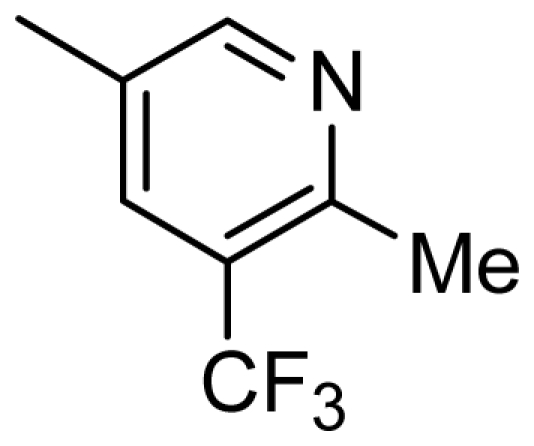	8.373	9.102
D5	Cl	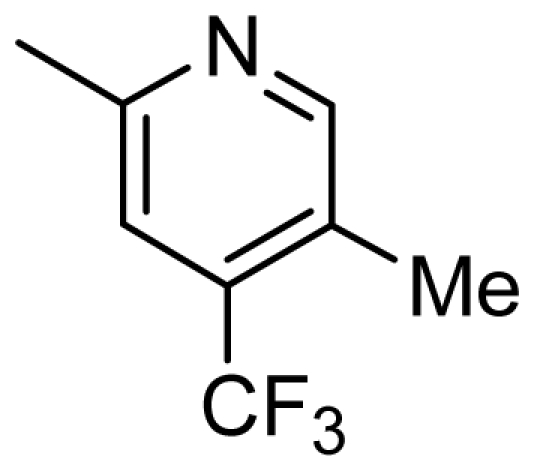	8.124	8.696
D6	Cl	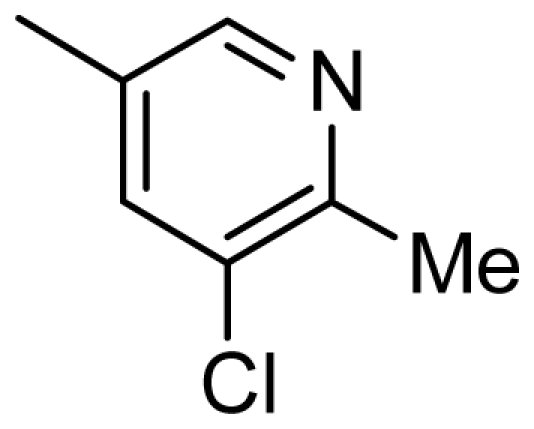	7.797	8.405
D7	H	3-EtPh	8.216	8.507
D8	H	3-CH_2_CF_3_Ph	8.378	8.891
D9	H	3,4-2CH_3_Ph	8.193	8.609
D10	H	3-CF_3_,4-CH_3_Ph	8.234	8.837
D11	Cl	3-CH_3_Ph	7.993	8.096
D12	Cl	3,4-2CH_3_Ph	8.170	8.582
D13	Cl	3-CF_3_,4-CH_3_Ph	8.258	8.814
D14	Cl	3-ClPh	7.898	8.245
D15	Cl	3-Cl,4-CH_3_Ph	8.191	8.731
